# The Higher Law of Medicine

**Published:** 1915-04

**Authors:** T. D. Crothers

**Affiliations:** Hartford, Conn.


### The Higher Law of Medicine.

BY T. D. CROTHERS, M. D., HARTFORD, CONN.

Medical students rarely hear anything, except the most materialistic conceptions of the laws of cause and effect, concerning the physical condition of the body.

When they enter upon practice they seem to be satisfied with the knowledge confined to medicine, and practically ignore all other topics or claim that they have no time to study them. Religion and theories of the world to come are considered as speculations, intangible and largely impractical.

When they come in contact with laymen and cultivated persons who have made a study of these higher facts of life their ignorance is painful, and they struggle to conceal it, and assume a loftier comprehension, based on coarse physical conditions. The mental side of medicine is an unknown country, except in the vaguest possible way.

Sometimes this great deficiency is repaired, and physicians become students of the higher realm of creative thought and thought energies. A large number of invalids suffer from perverted thoughts, ignorant reasoning and morbid fears that depress and destroy the vigor which they should possess. These are conditions that are not reached by medicines in any general way.

An example is that of a business man carrying great responsibilities and suffering from insomnia and nutrient disturbances, who visited many expert physicians, each of which made a physical diagnosis of organic troubles and prescribed for them.

Finally by accident he came in contact with a village physician, unknown to fame, who diagnosed his real condition. He found that he was suffering from concealed obsessions of death and disaster. This had started from a visit to a fortune teller, who told him that he would die at a certain time, suddenly, and unless he made an exact disposition of his wealth his family would become impoverished. At first he disbelieved this, but finally it became a settled conviction, and his anxiety to provide against this future brought on a nervous condition that simulated grave, organic diseases.

The physician talked with him every day, urged him to throw aside all drugs, take baths, healthy food and plenty of exercise, and by a process of mental suggestion and training followed up for a short time, restoration and a clearer conception of his duty to himself were brought about. Here was a new field in medicine.

The operation of these laws that are not taught in colleges are as veritable and far reaching as the effects of any drugs known. One of the first principles in this law is, that we create a large part of the disease and misery which we suffer from by abnormal thoughts and foolish misconceptions of the influence of surroundings. We become depressed by harboring depressing thoughts of weakness and incapacity, and this opens the door for bacterial invasion which resists the protective powers of the blood and vitality.

The man who is angry when conditions do not please him is cultivating a species of insanity and weakness that is certain to develop into organic disease.

The man who is continually worrying, struggling with doubts and fears, and anticipating failure, and never certain of success, is certain to have diseases of a peculiar type, because all his energies are reversed, and from this comes depression, lowered vitality, and absence of force. The man or woman who has but one idea in life, and that physical gratification, accumulation of property, and whose constant thought is how to obtain this are on the road and actually inviting diseases that are sure to come. Every man and woman carries about with them a certain thought atmosphere which is either attractive or repellant. They are injuring themselves by inability to concentrate their minds or energies along healthy lines and absorb from the surroundings the forces that make vigor and health. They are sufferers and die early.

Education that neglects all these higher mentalities is very often useless and destructive. The field of medicine that rises up into this realm and seeks to determine the energies that are building up or tearing down the brain and body, is not occupied by many persons, and is one of the grandest openings that science presents for the welfare of the race. Teaching men to live in the higher stories of their brain power, teaching them to recognize the surroundings and become adapted to them; that the real power of health and success in life is within and not without, is the highest truth in medicine; that all such terms as fate, surroundings and apparent adverse conditions are false theories and encourage disease and death.

The great fact should be reiterated over and over, that the laws of mind and creative thought force are as literal and exacting as those which govern the universe. Get in range with them, understand and apply them, and health, vigor and success are certain to follow. Ignore them, and act on other theories and conceptions, and disaster and failure are the result.

Cultivate the lower levels of hate, revenge, jealousy and avarice and these very elements are attracted and incorporated as the basal principles of life. These are the sidetracks that lead to disease and death. Cultivate the higher thoughts of hope, faith, love and charity, and these grow and ripen, and end in the fullest fruitage and flowerage of life. There are no creeds or religion in this. These are realities that the psychological studies of the present confirm in the largest way.

Among the few specialists in this field, whose activities are not heralded by journals, but felt in an increasing army of clients, are Dr. John Quackenbos of New York and Dr. Tom A. Williams of Washington, D. C. The former is a pioneer of many years standing. The latter is a psychologist who makes practical his theories by demonstrations.

There are other physicians in this field, but the work of these two men has come under my observation as a continual surprise and wonderment.

This field of psychic research is all about us, and white with the harvest, calling for competent men whose work will be far more startling and real than in any other field of medicine.

There are no spectacular shows in the hospital rooms to impress the public mind, but the quiet office consultations and the studies of the hidden thoughts, perverted reasons and reasonings of men and women everywhere opens up a field for prevention or correction that will bring in a new realm of practice.

Every physician in active practice recognizes the possibility of treatment in this field, and many others are actually giving assistance and help, both consciously and unconsciously, to their patients. The physician and teacher must be clear himself. He must know the abnormalities of thought and their effects on the system before he can help others to correct them.

